# Generalized Revenge

**DOI:** 10.1080/00048402.2019.1640323

**Published:** 2019-10-14

**Authors:** Julien Murzi, Lorenzo Rossi

**Affiliations:** University of Salzburg

**Keywords:** semantic paradoxes, paracomplete logics, paraconsistent logics, non-contractive logics, non-transitive logics, revenge paradoxes

## Abstract

Since Saul Kripke’s influential work in the 1970s, the revisionary approach to semantic paradox—the idea that semantic paradoxes must be solved by weakening classical logic—has been increasingly popular. In this paper, we present a new revenge argument to the effect that the main revisionary approaches breed new paradoxes that they are unable to block.

## 1. Introduction

Let λ be a sentence that says of itself that it is not true. On the plausible if naïve assumption that, for every sentence ϕ, ϕ and ‘ϕ is true’ are in some sense equivalent, a little reflection shows that λ is true if and only if it isn’t—a contradiction. In classical logic, this entails any sentence: that is, the reasoning makes one’s theory trivial. This is the Liar Paradox. Because the existence of sentences such as λ can be proved from basic syntactic principles, it is often thought that there are only two main ways out of the problem: one can either give up naïve principles about ‘true’ and other semantic notions, or revise classical logic. Since Saul Kripke’s influential work in the 1970s, the latter *revisionary* option has been increasingly popular.^1^ Authors such as Hartry Field have forcefully argued that the truth predicate plays a key expressive role in our cognitive lives—one that requires that ϕ and ‘ϕ is true’ be intersubstitutable. In a slogan, truth must be *naïve*.^2^ As a result, classical logic must be restricted on pain of triviality, but—revisionary theorists argue—this is not too high a cost, since classical principles are restricted where and only where they create trouble.^3^

Different non-classical theories of truth offer different explanations of the failure of classical principles. For instance, sentences that do not satisfy all of the principles of classical logic have been characterized as ‘paradoxical’ [Kripke 1975], ‘unstable’ [Zardini 2011], ‘indeterminate’ [McGee 1991; Field 2008], ‘glutty’ [Beall 2009], both ‘tolerantly assertible and deniable’ [Cobreros et al. 2013], and so on. In turn, these notions have been thought to give rise to specific *revenge arguments*: Liar-like reasonings aimed at showing that, while restricting certain classical principles allows non-classical theories to express a naïve notion of truth (and perhaps other semantic notions), notions such as absolute indeterminacy can only be expressed in those theories on pain of triviality.^4^ Revisionary theorists have responded by rejecting the coherence of revenge-breeding notions. For instance, Field writes that a unified notion of indeterminacy is ‘ultimately unintelligible’ [2008: 356]; similarly, Field, Jc Beall, and Graham Priest have rejected the coherence of the notion of *Boolean negation*.^5^ More generally, revisionary theorists typically dismiss semantic revenge arguments, on the ground that they assume (a non-instrumental reading of) classical semantics. However, revisionary theorists either reject classical semantics outright [Ripley 2013], or interpret it instrumentally,^6^ or argue that it should be no surprise that non-classical notions cannot be expressed from within a classical framework [Beall 2007a; Field 2008].

More recently, it has been argued that revisionary approaches validating the classical structural rules cannot express notions of *naïve validity* and that this fact should be taken to favour a *substructural approach*—one that restricts some of the classical structural rules (for discussion, see, for instance, Shapiro [2011a], Beall and Murzi [2013], Zardini [2014], Field [2017], and Murzi and Rossi [forthcoming]). Substructural approaches can express naïve truth, Boolean negation, and naïve validity [Zardini 2011; Ripley 2013; Nicolai and Rossi 2018]. Moreover, they have been argued to be ‘surprisingly strong’ and to approximate ‘the simplicity and symmetry of classical logic to an extent unmatched by its naive rivals’ [Zardini 2011: 512]. Indeed, David Ripley has argued in a number of papers that his favourite nontransitive logic of paradox just *is* classical logic.^7^

But are substructural approaches revenge immune? Is there a *general* revenge problem afflicting all kinds of revisionary approaches? In this paper, we present a new proof-theoretic revenge argument to the effect that the main revisionary approaches, structural and sub-structural alike, breed new paradoxes that they are unable to block. Our argument does not rely on semantic notions and, unlike existing revenge arguments, it applies in a uniform way to any minimally strong revisionary theory.

Our argument unfolds in two main stages. We start from the observation that current revisionary theories feature sentences such as t=t that satisfy all of the principles of classical logic in a given theory *S*, and sentences such as λ that satisfy such principles in *S* only on pain of triviality. We call sentences of the former kind *unparadoxical-in-S* and sentences of the latter kind *paradoxical-in-S*. We argue that these notions are perfectly intelligible, even by non-classical lights, and provide a general recipe for generating *revenge paradoxes* to the effect that the main revisionary theories can only be closed under naïve principles for paradoxicality and unparadoxicality on pain of triviality.

From a revisionary perspective, the most natural way out of the problem is to treat the new paradoxes in the same way as the paradoxes of truth—that is, by further weakening the logic. Since our revenge paradoxes rely on very weak logical resources, the upshot is that the revisionary approach is much more radical than it is usually thought to be.

To be sure, a more conservative reaction to the paradoxes of paradoxicality and unparadoxicality would be to question the intelligibility of these notions, much in the same way as notions such as absolute indeterminacy have already been questioned. However, we don’t think that ultimately this would do. For one thing, the distinction between paradoxical and unparadoxical sentences in our sense is a *simple fact* about revisionary theories: it is one that encodes a minimal lesson to be learned from the semantic paradoxes—namely, that if truth is naïve then sentences such as λ yield absurdity if reasoned with classically, while sentences such as t=t don’t (see, for example, Zardini [2011: 499]). For another, the distinction plays a crucial role in the main revisionary approaches to semantic paradox: it allows revisionary theories to ‘recapture’ classical theories such as classical mathematics, even if their logic is non-classical.

The plan of the paper is as follows. Sections 2–3 introduce the Liar Paradox and its four main revisionary ways out. Section 4 offers a precise definition of classical recapture. Section 5 presents four new revenge paradoxes, which trivialize the approaches introduced in section 4. Sections 6–7 discuss the relevance of our results and address potential objections. The proofs of our results are given in an Appendix.

## 2. The Liar Paradox

We begin with some technical preliminaries. Let $L_{Tr}$ be a first-order language with identity whose logical vocabulary includes ¬, ∧, ∨, →, ∀, and ∃. In addition,$\;L_{Tr}$ contains a propositional *absurdity* constant ⊥, a propositional *logical truth* constant ⊤, and a predicate Tr expressing truth. Terms and formulae of $L_{Tr}$ are defined as usual. Closed formulae are called ‘sentences’. We let t and s (possibly with indices) range over closed terms of$\;L_{Tr}$, and use ϕ, ψ, and χ (possibly with indices) as schematic variables for sentences of $L_{Tr}$.

We require that any theory that we consider satisfies two further requirements:

(i) There is a function ⌈⌉ such that, for every sentence ϕ, ⌈ϕ⌉ is a closed term. Informally, ⌈⌉ can be understood as a quote-name forming device, so that ⌈ϕ⌉ is a name of ϕ.

(ii) For every open formula ϕ(x) there is a term $t_{\phi }$ such that $\left\lceil \phi (t_{\phi }/x)\right\rceil$ is $t_{\phi }$, where $\phi (t_{\phi }/x)$ is the result of replacing every occurrence of x with $t_{\phi }$ in ϕ.

In order to satisfy (i) and (ii), a theory has to interpret a *modicum* of arithmetic or syntax theory. For simplicity, we only consider theories in which (i) and (ii) provably hold.

A *sequent* is an expression of the form Γ⊢ϕ, where Γ is finite *multiset* of sentences.^8^ The multiset to the left of ⊢ is the *antecedent* of a sequent; the sentence on the right of ⊢ is its *consequent*. We now recall the rules of classical propositional logic (henceforth CPL).^9^ Our axiomatisation is highly redundant, in order to simplify the definition of classical recapture to be given in section 4.^10^


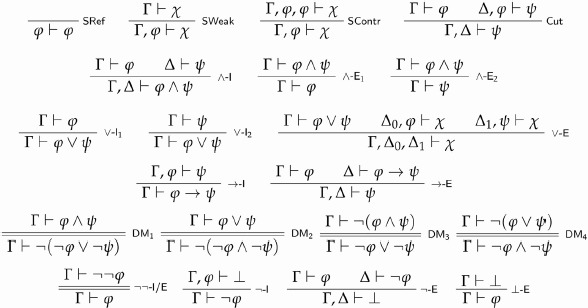


The sequents over the horizontal bar of a rule are its *premises*; the sequent below is its *conclusion*. A rule is an *inference* if its premises are empty, and a *meta-inference* otherwise.

In keeping with revisionary orthodoxy, we assume a *naïve* view of truth—that is, that the truth predicate satisfies the following truth rules (for convenience, we assume both positive and negative forms):


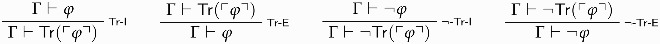


Other forms of naïveté include the T-Schema—Tr(⌈ϕ⌉)↔ϕ—and *transparency*—namely, the intersubstitutivity *salva veritate* of Tr(⌈ϕ⌉) and ϕ in all non-opaque contexts.

We are now in a position to present the Liar Paradox. Given our assumptions on $L_{Tr}$, we can prove that there is a sentence λ identical to ¬Tr(⌈λ⌉), so that λ says of itself that it isn’t true.^11^ We may then reason thus. We first prove Tr(⌈λ⌉)⊢⊥:


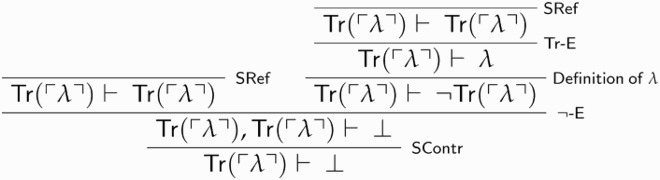


Call the above derivation $D_{0}$. We then derive Tr(λ) from $D_{0}$:


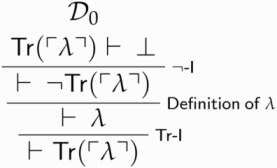


Call this derivation $D_{1}$. $D_{0}$ and $D_{1}$ can now be combined to yield a proof of absurdity, courtesy of Cut:


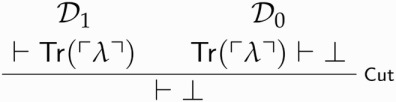


Given ⊥-E, it yields a proof of *any sentence ϕ*, thus trivialising any theory in which the paradox can be derived.^12^

## 3. Four Revisionary Ways Out

If naïve semantic principles such as Tr-I and Tr-E are non-negotiable, as revisionary theorists typically maintain, then one must blame the logic in order to avoid non-triviality. To be sure, such a revision is not to be taken lightly, and there is no shortage of classical treatments.^13^ But, contemporary logical wisdom has it, these alternatives are dire, the naïve semantic principles *are* non-negotiable, and there might be independent reasons for weakening classical logic in the first place.

The Liar Paradox makes use of four main logical ingredients: ¬-I, ¬-E, SContr, and Cut. Each of these rules can be, and indeed has been, questioned.^14^ We briefly consider the corresponding four revisionary strategies, and introduce, for each such strategy, the most representative corresponding formal theory.

### 3.1 Paracomplete and Paraconsistent

The most popular revisionary approaches to paradoxes such as the Liar involve revising the classical theory of negation and the conditional, according to which ¬ satisfies both ¬-I and ¬-E, and → satisfies both →-I and →-E. According to *paracomplete* theorists, sentences such as *λ* are *gappy*: they either lack a semantic value, or have an intermediate value between truth and falsity. According to *paraconsistent* theorists, sentences such as λ are *glutty*: that is, they are both true and false. We briefly review both approaches.^15^

Paracomplete theorists typically advocate the so-called *strong Kleene logic* K3 [Kleene 1952: 332–40], or some extension thereof. K3 is given by the rules of classical logic minus ¬-I and →-I. As a consequence, the Law of Excluded Middle (LEM)— ⊢ϕ∨¬ϕ—is not unrestrictedly valid either. We call K3TT the theory resulting from adding the naïve truth rules to a sufficiently expressive theory based on the logic K3.

Dually, paraconsistent theories are typically based on the logic LP, or some extension thereof [Asenjo 1966; Priest 1979]. LP is given by the rules of classical logic minus ¬-E and →-E. As a result, the Law of Non-contradiction—(LNC)ϕ∧¬ϕ⊢⊥—must be relinquished. We call LPTT the theory resulting from adding the naïve truth rules to a sufficiently expressive theory based on the logic LP.

### 3.2 Substructural Approaches: Non-Contractive and Non-Transitive

We now turn to approaches that restrict the *structural rules* SContr and Cut. *Non-contractive* approaches advocate a restriction of SContr. That is, according to these approaches, the fact that ψ follows from [ϕ,ϕ] does not entail that ψ follows from [ϕ] alone.^16^ Elia Zardini [2011] proves syntactic consistency for a transparent theory of truth whose underlying logic is a suitable strengthening of *multiplicative affine linear logic* (henceforth MALL). MALL’s propositional fragment is CPL without SContr and with ∨-E replaced by the following weaker version:


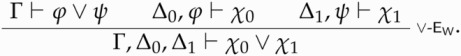


We call the propositional fragment of Zardini’s theory MALLTT, for a sufficiently expressive theory based on the logic MALL with transparent truth.^17^

Finally, *non-transitive* approaches recommend a restriction of Cut.^18^ In particular, Pablo Cobreros, Paul Egré, Robert van Rooij, and David Ripley have recently put forward a non-transitive theory based on the non-transitive logic ST, which is essentially classical logic, with all of its theorems and inferences, but without the rules Cut, →-E, ∨-E, and ¬-E. The theory, labelled STTT for *strict tolerant transparent truth*, allows for a uniform treatment of the semantic and indeed soritical paradoxes. For simplicity, we consider a sufficiently expressive theory of transparent truth, which we call $STTT_{0}$, given by a sub-logic of ST with the addition of the naïve truth rules. More precisely, the logic of $STTT_{0}$ is given by the rules of CPL minus Cut, →-E, ∨-E, and ¬-E.

## 4. Classical Recapture

The four families of non-classical theories that we have just introduced share a common feature: despite their non-classicality, they have *fully classical* fragments. That is, all of the theories presented in section 3 limit their restrictions to classical logic to *some* sentences. This is not only a basic fact about those theories; it also allows one to apply those theories to mathematics and science more generally. As is sometimes said, non-classical theories can *recapture* classical reasoning when needed.^19^ For instance, Field sees himself as being engaged in the project of finding [2008: 7]

a generalisation of classical logic that takes the classical rules to be appropriate for dealing with ‘ordinary’ predicates (such as those of standard mathematics and physics) but which allows only weaker rules when dealing with certain ‘extraordinary’ predicates [such as ‘true’].

Classical logic is restricted where, and only where, it creates trouble.^20^ To see how non-classical theories recapture classical theories, our starting point is a particularly simple way of characterising the classical fragment of K3TT, LPTT, MALLTT, $STTT_{0}$, and their extensions. Such theories enjoy the following informal property:

**(Classicality Principles)** There are finitely many classically valid principles such that a sentence satisfies such principles only if it satisfies all classical principles.

We can then say that a theory recaptures classical logic if it is closed under weaker versions of classical rules that, whenever some extra conditions are satisfied, reduce to their classical counterparts. The following definition formally captures this idea.

**Definition 4.1** (Classical recapture)**.** Let *S* be a non-trivial theory. Then *S* enjoys a *classical recapture property* if it is P-classical recapturing, for some classically valid principle P invalid in *S*. The following classical recapture properties correspond to the revisionary approaches reviewed in sections 3.1–3.2.

- *S* is LEM*-classical recapturing* if it is closed under the rules of CPL, where →-I and ¬-I are replaced by the following weaker versions:
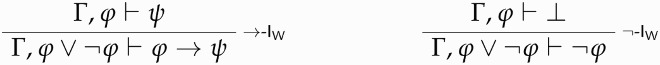
- *S* is LNC-*classical recapturing* if it is closed under the rules of CPL, where →-E and ¬-E are replaced by the following weaker versions:^21^
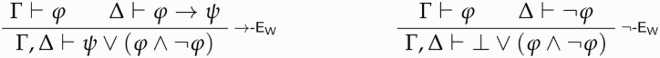
- *S* is LContr-*classical recapturing*,
  (LContr)⊢ϕ→(ϕ∧ϕ),

if it is closed under the rules of CPL, where SContr is replaced by the following weaker version:


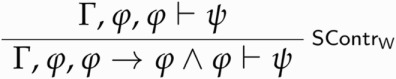

- *S* is Cut*-classical recapturing* if it is closed under the rules of CPL *minus* Cut, where →-E, ¬-E, and ∨-E are replaced by the following weaker versions:
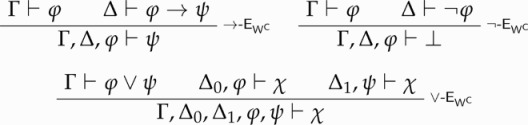

We now show that classical logic can be recaptured in the sense of Definition 4.1, in each of the non-classical approaches introduced in section 3. We do so by adding the classical recapturing rules to our target theories, and then establishing that classical logic holds for *ϕ* whenever the relevant classical principles hold for ϕ.

**Definition 4.2** ($K3TT^{+}$)**.** $K3TT^{+}$ is the result of adding →-$I_{W}$ and ¬-$I_{W}$ to K3TT.

By definition, $K3TT^{+}$ is LEM-classical recapturing. To see that full CPL holds for ϕ in $K3TT^{+}$ given ϕ∨¬ϕ, it is sufficient to notice that, whenever ϕ∨¬ϕ is derivable in $K3TT^{+}$, both →-I and ¬-I hold in $K3TT^{+}$. More precisely, if Γ,ϕ⊢⊥ is derivable together with ϕ∨¬ϕ, then we can apply ¬-$I_{W}\;$and apply Cut to ϕ∨¬ϕ, thus deriving Γ⊢¬ϕ—that is, the conclusion of full ¬-I. Similarly for →-I.

**Definition 4.3** ($LPTT^{+}$)**.** $LPTT^{+}$ is the result of adding →-$E_{W}$ and ¬-$E_{W}$ to LPTT.

By definition, $LPTT^{+}$ is LNC-classical recapturing. As above, in order to see that full CPL holds for ϕ in $LPTT^{+}$ if ϕ∧¬ϕ⊢⊥ does, it is sufficient to notice that full →-E and ¬-E hold for ϕ in $LPTT^{+}$ whenever ϕ∧¬ϕ⊢⊥ is derivable in $LPTT^{+}$. More precisely, if Γ⊢ϕ and Δ⊢ϕ→ψ are derivable together with ϕ∧¬ϕ⊢⊥, one can derive Γ,Δ⊢ψ courtesy of →-$E_{W}$ and ∨-E. At a glance:


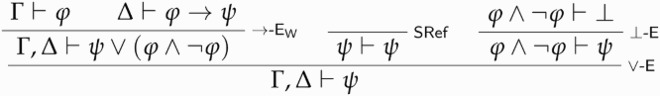


The reasoning for ¬-E is analogous.

**Definition 4.4** ($MALLTT^{+}$)**.** $MALLTT^{+}$ is the result of adding $SContr_{W}$ to MALLTT.

As above, by definition $MALLTT^{+}$ is LContr-classical recapturing. To see that classical logic holds for ϕ if LContr holds for ϕ, we reason in two steps, keeping in mind that MALL is classical logic minus SContr and with ∨-E replaced with ∨-$E_{W}$.^22^ First, it is immediate to see that whenever LContr holds for ϕ, then SContr also holds:


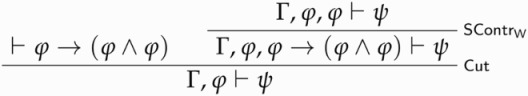


Second, we show that RContr —that is, ⊢(ϕ∨ϕ)→ϕ—is derivable from SContr and that, in turn, ∨-E is derivable from RContr. The following derivation establishes the first claim:


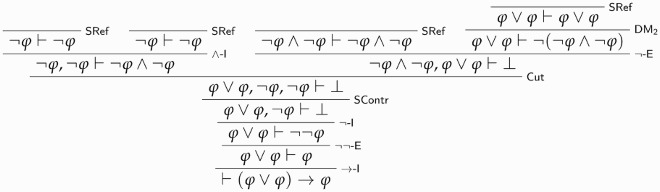


The second claim is proved as follows:


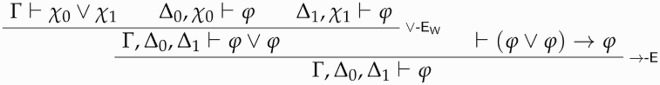

**Definition 4.5** (${STTT}_{0}^{+}$)**.** ${STTT}_{0}^{+}$ is the result of adding →-$E_{W^{C}}$ , ¬-$E_{W^{C}}$, and ∨-$E_{W^{C}}$ to $STTT_{0}$.

By definition, ${STTT}_{0}^{+}$ is Cut-classical recapturing. It can be verified that, whenever Cut holds for ϕ, full classical logic holds for ϕ. To see this, consider →-E and suppose that Cut holds for ϕ. Then, given ⊢ϕ, full →-E is derived as follows:


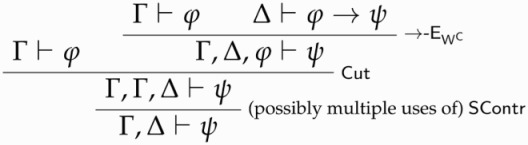


A similar reasoning applies to ¬-$E_{W^{C}}$ and ∨-$E_{W^{C}}$.^23^

The classical recapturing properties of the non-classical theories introduced in section 3 are at the heart of our general revenge argument, to which we now turn.

## 5. Revenge

Revenge arguments fall into two broad categories—*object-linguistic* and *meta-theoretic*.^24^ Meta- theoretic revenge arguments point to the inexpressibility in a theory *S* of notions definable in *S*’s meta-theory (which is typically classical). They are standardly dismissed on the ground that it is no surprise that classical notions are not expressible in a non-classical theory.^25^ Object-linguistic revenge arguments typically point to the inexpressibility in a theory *S* of some notion *N* that plays some explanatory or expressive role in *S*. Notions such as indeterminacy [Field 2007, 2008] and instability [Zardini 2011] are cases in point.^26^ The revenge paradoxes to be developed in this section are of the object-linguistic kind. In particular, they don’t rely on classical semantic notions, and they apply to theories (such as the one developed by Zardini [2011]) for which no semantics is known. Sections 5.1–5.2 motivate naïve principles for paradoxicality and unparadoxicality. Sections 5.3–5.6 introduce our revenge paradoxes.

### 5.1 Paradoxicality and Unparadoxicality

General approaches to revenge are discussed by Beall [2007c], Priest [2007], Shapiro [2011b], and Scharp [2013, sec. 4.3]. For instance, Priest [2007: 226] argues that

[t]here is, in fact, a uniform method for constructing the revenge paradox—or extended paradox, as it is called sometimes. All semantic accounts have a bunch of Good Guys (the true, the stably true, the ultimately true, or whatever). These are the ones that we target when we assert. Then there’s the Rest. The extended liar is a sentence, produced by some diagonalising construction, which says of itself that it’s in the Rest. The diagonal construction … may then play havoc. This shows, incidentally, that the extended paradox is not really a different paradox. The pristine liar is the result of the construction when the theoretical framework is the standard one (all sentences are true or false, not both, and not neither) … ‘Extended paradoxes’ are simply the results of applying the construction in different theoretical frameworks.

We are sympathetic to Priest’s claim that revenge paradoxes are structurally similar to the run-of-the-mill semantic paradoxes. However, his revenge recipe only describes extremely general features of revenge arguments, and cannot be used to actually generate, in a uniform way, revenge paradoxes for a wide range of theories. Our aim in what follows is to provide a general revenge strategy for constructing revenge paradoxes for several non-classical theories satisfying **Classicality principles**, including some of the non-classical theories defended by Priest.

Our starting point is the distinction, present in each of the theories presented in section 3, between sentences that satisfy all of the principles of classical logic and sentences that do so on pain of triviality. More precisely, let *S* be a $P_{0}$, … , $P_{n}$-classical recapturing, non-trivial theory. We then say that a sentence ϕ is *paradoxical-in-S* if and only if ⊥ follows in *S* from the assumption that ϕ satisfies $P_{0}$, … , $P_{n}$; and that a sentence ϕ is *unparadoxical-in-S* if and only if it satisfies $P_{0}$, … , $P_{n}$in *S*.^27^ Paradoxicality and unparadoxicality, so understood, are intelligible notions at the core of the revisionary approach to semantic paradox. In keeping with the revisionist’s treatment of truth, we treat them as *object-linguistic predicates*, Par and Un.^28^

### The Expressive Role of Par and Un

5.2

Revisionary theorists typically give the semantics of a language L *in* L. Here are two representative quotes:

If the formal language is to provide an adequate explication of the informal language that we use, it must contain its own metalanguage. [Reinhardt 1986: 227–9]

my claim will be that there are languages that are sufficiently powerful to serve as their own meta-languages. [Field 2008: 18]

They further argue that the notion of truth for L to be captured in L must be *naïve*.^29^ For instance, the following reasoning is taken to motivate the unrestricted rule Tr-E:

**Agreement.** All of the theorems of Peano Arithmetic are true. ϕ is a theorem of Peano Arithmetic. Therefore, ϕ is true. Therefore, ϕ.

A parallel reasoning is taken to establish Tr-I:

**Disagreement.** Everything that Lois says is not true. Lois says ϕ. Therefore, ϕ is not true. Therefore, ¬ϕ.

Similar considerations can be put forward for paradoxicality and unparadoxicality. For reasons of space, we only give one example, in the context of a paracomplete theory of naïve truth *S*. Consider the following case:

**The logic student.** Lois is a logic student who is learning how to reason in *S*. She (mistakenly) assumes λ∨¬λ. As a result, she carries out the Liar reasoning in *S* and derives ⊥. She concludes that assuming that λ satisfies LEM trivializes *S*. As she puts it, λ is paradoxical: that is, Lois asserts Par(⌈λ⌉).

In the above example, Lois adopts the following principle—namely, that if *S* derives the sequent ϕ∨¬ϕ⊢⊥ then it also derives the sequent ⊢Par(ϕ). We call this principle Par- introduction (Par-I, for short). The principle immediately rules out the possibility of interpreting paradoxicality by means of a conditional. That is, given Par-I, Par(x) cannot be interpreted as (slightly abusing notation) Tr(x∨¬x)→⊥, since, in a paracomplete setting, →-I is not unrestrictedly valid and, as a result, Tr(⌈λ∨¬λ⌉)→⊥ cannot in general be inferred from a derivation of ⊥ in *S* from λ∨¬λ.

It might be tempting to interpret Par instead as derivability in *S*. After all, if *S* interprets a *modicum* of arithmetic, if there is derivation in *S* of ⊥ from λ∨¬λ, then *S* derives $Der_{S}(\left\lceil \lambda \vee \neg \lambda \rceil ,\;\;\lceil ⊥\right\rceil )$, where $Der_{S}$ is a standard, arithmetically definable, derivability predicate for *S*. However, this can’t be either, as shown by the following scenario:

**Misguided reasoning.** Clark reasons in *S* and assumes that everything that Lois says is paradoxical. Lois asserts that ϕ. As a result, Clark infers that ϕ is paradoxical. However, Clark also proves that ϕ satisfies LEM, and hence all of the principles of classical logic. From his claim that ϕ is paradoxical (that is, such that ϕ∨¬ϕ entails ⊥), and his proof of ϕ∨¬ϕ, Clark concludes ⊥.

The above scenario requires the following elimination rule: from Par(⌈ϕ⌉) and ‘ϕ satisfies LEM’, one may infer ⊥. We call this principle Par-elimination (Par-E, for short). Just as Par-I rules out interpreting paradoxicality by means of a conditional, Par-E rules out interpreting such a notion as derivability-in-*S*. This is essentially a consequence of Löb’s Theorem, as we will see more fully in section 6.1.

We conclude that paradoxicality-in-*S* must be expressed via a single primitive predicate Par, obeying Par-I and Par-E. Similar arguments apply to unparadoxicality-in-*S*, and to paraconsistent, non-contractive, and non-transitive theories.

The notions of paradoxicality and unparadoxicality now give rise to a *revenge argument*, to the effect that any theory extending the theories presented in section 3 expresses such notions only if it is trivial. In particular, consistent theories cannot express the notion of paradoxicality, while inconsistent theories cannot express the dual notion of unparadoxicality. We consider theories formulated in the language $L_{Tr}^{+}$obtained by adding Par and Un to $L_{Tr}$. We extend to $L_{Tr}^{+}$, and the theories formulated in it, all of the conventions and requirements stated in section 2 for languages and theories.

### 5.3 Paracomplete Revenge

We focus on $K3TT^{+}$-based theories as our representative, catch-all, paracomplete theories. Since paracomplete theories reject LEM for ‘paradoxical’ sentences, and since $K3TT^{+}$is LEM-classical recapturing, the rules for Par are as follows:^30^


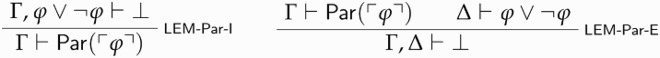

**Definition 5.1** (K3TTP)**.** K3TTP is the theory resulting from closing $K3TT^{+}$ under LEM-Par-I and LEM-Par-E.

**Proposition 5.2.** K3TTP *is trivial, and so is the closure under* LEM-Par-I *and* LEM-Par-I *of any theory extending* $K3TT^{+}$ *.*

It follows from Proposition 5.2 that (among others) the theories developed by Field [2002, 2008, 2013] and Yablo [2003] cannot express the notion ‘ϕ yields absurdity if ϕ∨¬ϕ holds’, on pain of triviality.

### 5.4 Paraconsistent Revenge

Consider paraconsistent approaches. In keeping with our account of classical recapture, we focus on $LPTT^{+}$-based theories. We show that no extension of $LPTT^{+}$ can express the notion of *unparadoxicality* introduced in section 5.1. Keeping in mind that $LPTT^{+}$ is LNC-classical recapturing, a sentence ϕ is unparadoxical in $LPTT^{+}$ if LNC holds for ϕ—that is, if $LPTT^{+}$ proves ϕ∧¬ϕ⊢⊥. Conversely, if ϕ is unparadoxical in $LPTT^{+}$, then LNC holds for ϕ: that is, if $LPTT^{+}$ proves ϕ∧¬ϕ from Γ, Δ, then it also proves ⊥ from the same multi-set of assumptions. More formally:


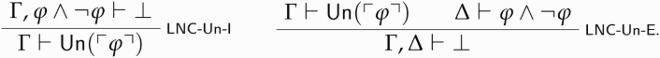

**Definition 5.3** (LPTTU)**.** LPTTU is the theory resulting from closing $LPTT^{+}$ under LNC-Un-I and LNC-Un-E.

**Proposition 5.4.** LPTTU *is trivial, and so is the closure under* LNC-Un-I *and* LNC-Un-E *of any theory extending* $LPTT^{+}$ *.*

It follows from Proposition 5.4 that the theories developed by Priest [2006b] and Beall [2009, 2011] cannot express the notion ‘ϕ behaves classically if ϕ∧¬ϕ⊢⊥ holds’, on pain of triviality.

### 5.5 Non-Contractive Revenge

Now to contraction-free approaches. Because of its prominence, we focus on Zardini’s non-contractive theory, but our result generalizes. We begin by recalling classical recapture in a contraction-free setting. As we have seen in section 4 (Definition 4.4 and subsequent remarks), full SContr and ∨-E, and hence full classical logic, hold for ϕ in $MALLTT^{+}$whenever $MALLTT^{+}$ derives ϕ→(ϕ∧ϕ). Keeping in mind that, according to SContr-free wisdom, SContr is the culprit of the semantic paradoxes, the paradoxicality predicate can now be interpreted as follows: if absurdity is derivable from the assumption that *ϕ* satisfies ϕ→(ϕ∧ϕ), then ϕ is paradoxical. Conversely, if ϕ is paradoxical and ϕ satisfies ϕ→(ϕ∧ϕ), then ⊥ is derivable.

This informal reasoning can be formalized thus. Let $[\phi {]}^{n}$ be the multiset consisting of *n* occurrences of ϕ. Moreover, let us assume that Γ in LC-Par-I does not contain instances of ϕ→(ϕ∧ϕ), and let *m* ≥ 1. Then paradoxicality in a non-contractive setting is characterized by the following rules:


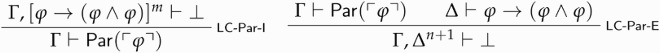


where *n* is the highest number of occurrences of ϕ→(ϕ∧ϕ) occurring on the left-hand side of the sequents in the subderivation of Γ⊢Par(⌈ϕ⌉) if Γ is non-empty, and 0 otherwise. Intuitively, the I-rule tells us that if contracting *m* times on ϕ yields absurdity (where *m* contractions on ϕ are represented by [ϕ→(ϕ∧ϕ)]^*m*^), then ϕ is paradoxical. Conversely, the E-rule says that if ϕ is paradoxical then the assumption that ϕ can be contracted on (at least as many times as are needed to declare it paradoxical) yields absurdity.

**Definition 5.5** (MALLTTP)**.** MALLTTP is the theory resulting from closing $MALLTTP^{+}$ under LC-Par-I and LC-Par-E.

**Proposition 5.6.** MALLTTP *is trivial, and so is the closure under*  LC-Par-I *and*  LC-Par-E *of any theory extending* $MALLTTP^{+}$ *.*

It follows from Proposition 5.6 that (among others) the theory developed by Zardini [2011] cannot express the notion ‘ϕ yields absurdity if [$\phi \to (\phi \wedge \phi ){]}^{m}$ holds’, on pain of triviality.

It might be objected that the non-contractive theorist who rejects contraction *in all of its forms* has a reason to reject contracting on sentences of the form ϕ→(ϕ∧ϕ), and hence to reject LC-Par-I, which allows one to discharge multiple occurrences of ϕ→(ϕ∧ϕ). Rather, the non-contractive theorist might insist that there exist denumerably many notions of paradoxicality, depending on how many times the assumption *ϕ* → (*ϕ* ∧ *ϕ*) is needed in order to derive ⊥. For instance, if ⊥ is derivable from just one copy of *ϕ* → (*ϕ* ∧ *ϕ*), then *ϕ* is paradoxical_1_; if it is derivable from two such copies, then it is paradoxical_2_; and so on. Then, the noncontractive theorist might point out, the proof of Proposition 5.6 breaks down, since it equivocates between paradoxicality_2_ (introduced on line 3 of derivation D_1_) and paradoxicality_5_ (introduced on line 3 of derivation D_2_) – see the Appendix for the proofs. However, the resulting conception of paradoxicality would be highly problematic. It would commit the non-contractive theorists to infinitely many notions of paradoxicality, which would sit poorly with her diagnosis of what goes wrong in paradoxical derivations. According to non-contractive wisdom, *indiscriminate uses* of SContr must be rejected *in general*. That is, non-contractive theorists disallow the following generalized version of SContr:


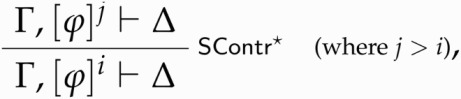


according to which, if Δ follows from Γ and *i* occurrences of ϕ, then Δ follows from Γ and at least one occurrence of ϕ. The idea that if $SContr^{*}$ applied to ϕ leads to ⊥ then ϕ is non-contractable is at the heart of the non-contractive approach to semantic paradox: one must disallow *whatever number* of applications of SContr to ϕ lead to ⊥ in a paradoxical derivation. This is captured by our rule LC-Par-I, but cannot be expressed by the non-contractive theorist who expresses paradoxicality by means of denumerably many paradoxicality predicates.

### 5.6 Non-Transitive Revenge

Finally, we turn to non-transitive approaches. We focus on the theory ${STTT}_{0}^{+}$ but, again, our results generalize. To begin, we notice that, in ${STTT}_{0}^{+}$, full classical logic holds for ϕ whenever Cut does (see Definition 4.5 and subsequent remarks). This in turn justifies the following characterisation of unparadoxicality. On the one hand, if ϕ is ‘cuttable on’—that is, if the conclusion of an instance of Cut applied to ϕ is derivable from its premises—then ϕ is unparadoxical. On the other, if ϕ is unparadoxical and the premises of an instance of Cut applied to ϕ are derivable, so is their conclusion.

Our revenge argument against ${STTT}_{0}^{+}$ shows that such a theory cannot express un- paradoxicality, so understood. It makes use of *higher-order* rules—rules that allow one to discharge *entire sequents*, as well as sentences.^31^ We are now in a position to formulate the rules governing the unparadoxicality predicate:


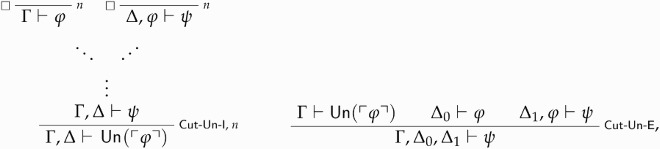


where the box left of the discharge line in Cut-Un-I signals that the rule-assumptions Γ⊢ϕ and Δ, ϕ⊢ψ may not be discharged vacuously.^32^ Again, the rules are justified by the account of classical recapture given in section 4 (see, especially, Definition 4.5). Cut-Un-I says that if ϕ is ‘cuttable on’ then it is unparadoxical. Conversely, Cut-Un-E tells us that if ϕ is unparadoxical (given Γ), and hence ‘cuttable’, and both $\Delta_{0}\;⊢\;\phi$ and $\Delta_{1}$, ϕ⊢ψ are provable, then Γ, $\Delta_{0}$, $\Delta_{1}⊢\psi$ follows.

**Definition 5.7** ($STTTU_{0}$)**.** $STTTU_{0}$ is the theory resulting from closing ${STTT}_{0}^{+}$ under Cut-Un-I and Cut-Un-E.

**Proposition 5.8.** $STTTU_{0}$ *is trivial, and so is the closure under* Cut-Un-I *and* Cut-Un-E *of any theory extending* ${STTT}_{0}^{+}$ *.*

It follows from Proposition 5.8 that (among others) the theories developed by Ripley [2012] and Cobreros et al. [2013] cannot express the notion ‘*ϕ* behaves classically given a derivation of ⊢ψ from ⊢ϕ and ϕ⊢ψ’, on pain of triviality.

We notice that the derivation of ⊥ in the proof of Proposition 5.8 (see Appendix) is not normal, since it involves a use of Cut-Un-E immediately after a use of Cut-Un-I.^33^ This suggests that, unlike ${STTT}_{0}^{+}$, Neil Tennant’s *Core Logic*, a logic in which all proofs are normal proofs, may support the rules for naïve truth *together with* Cut-Un-I and Cut-Un-E, in keeping with Tennant’s conjecture that the semantic paradoxes all involve derivations that cannot be brought into normal form [1982, 2015].^34^ Does it follow that our revenge argument doesn’t apply to the non-transitive approach defended by Tennant [2015]? He advocates a positive answer [ibid.: 593]. However, we do not share his optimism. In the proof of Proposition 5.8, we give *normal* proofs of ⊢Un(⌈ς⌉), ⊢ς, and ς⊢⊥, where ς is ¬Tr(⌈ς⌉)∧Un(⌈ς⌉). That is, Tennant’s theory proves both that ϕ is ‘cuttable’ and the premises of a cut on ϕ. Yet *one cannot cut on* ϕ in such a theory. This means that Un(⌈ς⌉)—that is, that ϕ is ‘cuttable’, no longer has its intended meaning in Tennant’s framework. The framework is not trivial, but non-triviality is restored only at the price of expressive incompleteness.

## 6. What Our Results Show

The paradoxes of sections 5.3–5.6 make use of logical rules that are valid in the theories they trivialize. In so far as the theories introduced in section 3 are representative of the revisionary approach to semantic paradox, it follows that revisionary treatments of the Liar Paradox and of other run of the mill paradoxes don’t apply to the paradoxes in sections 5.3–5.6. Yet the notions of paradoxicality and unparadoxicality codify a minimal lesson to be drawn from the semantic paradoxes—that, given the naïve truth rules, sentences such as λ satisfy all of the classical rules only on pain of triviality, whereas sentences such as *t* = *t* unproblematically satisfy those rules. The results of sections 5.3–5.6 show that the expression of such a truism is precluded to most non-classical theorists, on pain of adopting an extremely weak, and possibly unworkable, logic. For instance, it is a consequence of the proof of Proposition 5.2 that a paracomplete logic of paradox cannot contain all of SRef, SContr, →-E, and ∨-I. Likewise, it follows from the proof of Proposition 5.8 that a non-transitive logic of paradox cannot contain SRef, SContr, the rules for conjunction, and a very weak form of negation elimination. And so on. Our revenge strategy is perfectly general. Although the paradoxes of sections 5.3–5.6 make use of theory-specific notions of paradoxicality and unparadoxicality, it can be shown that the naïve rules for Par and Un are instances of a more general template.^35^

Solomon Feferman [1984: 95] once wrote, referring to theories of truth based on the logic K3, that ‘nothing like sustained ordinary reasoning can be carried out’ in them. While his remark might apply to weak logics such as K3 and LP, it may be thought to be unfair as a criticism of the stronger non-classical theories developed since 1984, such as the structural ones given by Field [2002, 2008, 2017] and Beall [2009], and the substructural ones given by Zardini [2011] and Cobreros et al. [2012]. Even classical theorists concede that, *pace* Feferman, some such theories are surprisingly strong. Vann McGee [2010], for instance, reports to have been ‘astonished’ by the ‘combination of transparency and logical strength’ exhibited by Field’s paracomplete theory.

The results of sections 5.3–5.6 vindicate the spirit of Feferman’s remark. Just as classical logic, and many other strong logics, are known to be incompatible with naïve truth, our results show that a wide range of reasonably strong non-classical logics are incompatible with naïve paradoxicality and unparadoxicality. And, as we argued in sections 5.1–5.2, just as there are strong reasons for wanting truth to be naïve, and hence to adopt one of the non-classical logics introduced in section 4 (or some extension thereof), there are parallel reasons for wanting paradoxical and unparadoxicality to *also* be naïve, and hence to adopt an even weaker non-classical logic—one in which the arguments of sections 5.3–5.6 no longer go through. By the revisionary theorist’s own lights, strong non-classical theories such as Field’s are ultimately incompatible with the project of giving the semantics of a language L in L.

In what follows, we briefly explore the relation between our results and Löb’s Theorem (section 6.1). We argue that the naïve principles for paradoxicality and unparadoxicality can be seen to be compatible with classical limitative results such as Löb’s Theorem, in just the same way as a naïve notion of truth can be seen to be compatible with classical limitative results such as Tarski’s Theorem. We then point to a parallel between our arguments and a recent revenge argument for classical theories (section 6.2).

### 6.1 Paradoxicality and Derivability

It could be argued that the results in sections 5.3–5.6 are hardly surprising, on the ground that the eliminations rules for Par and Un are unacceptable in the lights of Löb’s Theorem. More precisely, let *S* be a theory satisfying the Hilbert-Bernays conditions for a predicate $Prov_{S}$ expressing standard provability-in-*S*.^36^ It is a consequence of Löb’s Theorem that, if *S* proves every instance of $Prov_{S}(\lceil \phi \rceil )\to \phi$, then it also proves any sentence ϕ. Consider the paradoxicality predicate Par. Its rules can be rewritten, using a two-place derivability predicate $Der_{S}(x,\;y)$ expressing that *y* is derivable from *x* in *S*. For instance, the LEM-Par rules can be seen as instances of the following general rules:


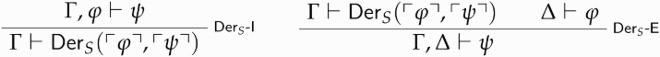


However, $Der_{S}$-E entails $Der_{S}(⊤,\lceil \psi \rceil )\to \psi$, which is equivalent to $Prov_{S}(\lceil \psi \rceil )\to \psi$, from which ψ is derivable in *S* via Löb’s Theorem. It is now natural to object that the rules for Par and Un employed by the results in sections 5.3–5.6 are but special cases of naïve rules for provability-in-*S* that are *already known* to be unacceptable because of Löb’s Theorem.^37^

The foregoing reasoning requires that paradoxicality-in-*S* be interpreted as standard derivability-in-*S*. More precisely, it assumes that the paradoxicality and unparadoxicality predicates be interpreted by means of an arithmetically definable derivability predicate $Der_{S}$ satisfying versions of the Hilbert-Bernays derivability conditions. On such a construal, the introduction rules for Par and Un are arithmetically derivable, while the elimination rules hold only on pain of triviality. However, we have argued in 5.2 that Par is *not* to be interpreted via a standard derivability predicate: scenarios such as **Misguided reasoning** rule out this possible interpretation.

If it is insisted that paradoxicality and unparadoxicality are to be interpreted via a standard derivability predicate, and therefore fail to obey their elimination rules because of Löb’s Theorem, then a parallel argument can be given that *truth* is to be interpreted via some arithmetically definable predicate, and therefore fails to obey the naïve truth rules, because of Tarski’s Theorem. For instance, it might be pointed out that sufficiently strong theories validate all instances of the T-Schema restricted to $\Sigma_{n}$-sentences of the base language. More precisely, they validate all instances of the following schema: $\phi ↔{Tr}_{\Sigma_{n}}(\lceil \phi \rceil )$, for ϕ a $\Sigma_{n}$-sentence of the base language (for any given *n*) and $Tr_{\Sigma_{n}}$ a predicate definable in the base theory. To be sure, restricting the T-Schema to $\Sigma_{n}$-sentences is inadequate for the purpose of giving the semantics of a language L in L.^38^ However, it might be argued, the same holds for any restricted notion of paradoxicality or unparadoxicality. For instance, if Par-I is restricted, some sentences that behave non-classically in *S* cannot be said to be paradoxical and, if Par-E is restricted, one cannot infer from the claim that ϕ is paradoxical that ϕ satisfies *S*’s classical recapturing principles only on pain of triviality. Just as it is possible to validate all instances of the T-Schema in spite of Tarski’s Theorem, it is also possible, and consistent with Löb’s Theorem, to non-trivially have all instances of the naïve rules for paradoxicality and unparadoxicality, provided that one adopts a *very weak* non-classical logic.

### 6.2 Non-Classical and Classical Revenge

The revenge paradoxes for non-classical theories given in sections 5.3–5.6 are closely related to a general revenge argument for classical theories recently introduced by Andrew Bacon [2015]. Bacon’s starting point is analogous to ours: while non-classical theories of truth restrict the application of classical logic to some sentences, classical theories of truth restrict the application of *naïve truth-theoretic principles* to some sentences.^39^ In order to express such a distinction, Bacon introduces a ‘healthiness’ predicate H satisfying the following scheme:

(SRT)H(⌈ϕ⌉)→(Tr(⌈ϕ⌉)↔ϕ).

That is, Bacon characterizes the healthy sentences as those that satisfy naïve truth-theoretic principles such as the T-Schema. Bacon then shows that every (sufficiently expressive) classical theory of truth that includes all instances of SRT proves sentences that it also proves to be unhealthy—that is, proves ϕ∧¬H(⌈ϕ⌉) for some ϕ. Under the assumption (which Bacon does not endorse) that H satisfies the following necessitation rule


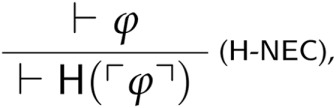


Bacon’s argument shows the resulting theories to be trivial.

The parallel between Bacon’s argument and ours is easy to see. On one hand, his argument shows that *classical* theories cannot be closed under natural principles governing a healthiness predicate true of all and only the sentences that satisfy the naïve truth rules, keeping classical logic fixed (and similarly for unhealthiness). On the other hand, our results show that *non-classical* theories cannot be closed under natural principles governing a unparadoxicality predicate, true of all and only the sentences that satisfy all of the principles of classical logic, keeping the naïve truth rules fixed (and similarly for paradoxicality).

## 7 Objections and Replies

Revisionary theorists might object to the paradoxes in sections 5.3–5.6 on the ground that our naïve principles for paradoxicality and unparadoxicality trade on a deep misunderstanding of their views. More specifically, they might argue that our revenge arguments try to force revisionary theories to express notions against whose intelligibility they have long argued.^40^ For instance, Field [2008: 309] writes that

there is no negation that obeys [both of ¬-I and ¬-E] without restriction: if there were, it would be impossible to have a [naïve] truth predicate.

He further suggests that there is no coherent notion satisfying both →-I and →-E. Similarly, it might be argued that, while the non-classical theories of section 3 cannot express paradoxicality or unparadoxicality, this is not a problem, since there is no coherent notion to be expressed beyond the ones already expressible in such theories. For instance, the non-contractive theorist might insist that LC-Par-I validates some illicit, and ultimately unacceptable, uses of contraction. Likewise, the non-transitive theorist might insist that ‘cuttable’ is to be interpreted by means of a conditional: *if* one can assert the premises of a cut on ϕ, *then* one may assert the conclusion of such a cut. She might then point out that to assume that one can in general infer the consequent of this conditional from the premises is just to assume the unrestricted validity of →-E, which non-transitive theorists reject (since it makes Cut admissible).

This kind of reply is perfectly coherent, as far as it goes. But how far does it go? As we observed in section 5.5, LC-Par-I expresses the basic non-contractivist diagnosis of the paradoxes—namely, that *contraction in general* is at the root of those paradoxes. Whether *S* is trivialized by one, two, or *m* uses of contraction, these are manifestations of the *same* problem. Similarly, if one’s logic doesn’t allow interpreting ‘cuttable’ in such a way that one *can* cut on a cuttable sentence, then this is a serious expressive limitation of the logic. The English expression ‘cuttable’ still means cuttable, and any adequate solution to the paradoxes should respect this basic fact about English. (Imagine the surprise of our logic student, Lois, if she were to learn that, even if ϕ is cuttable and one can assert the premises of a cut on ϕ, one might still not be allowed to derive the conclusion of such a cut.) Similarly for the other cases: rejecting the rules for Par and Un restores non-triviality only at the price of serious expressive limitations.

We can think of two main possible reactions at this point. First, upon deriving ⊥ in *S* from a classical recapturing principle, non-classical theorists might concede that ϕ *is* paradoxical, and insist that it is just a limitation about *S* that it cannot non-trivially prove as much—a limitation with which one must learn to live. However, while classical theorists may be sympathetic to this suggestion, it does not sit well with the project of giving the semantics for a language L *in* L.

Second, one might offer instead a hierarchical treatment of the notions of paradoxicality and unparadoxicality, much in the same way as, in order to semantically characterize intuitively defective sentences such as λ, Field [2007, 2008: chs. 22–3] defines a hierarchy of determinacy operators.^41^ For instance, the thought would be that, although the sentence *ρ* identical to Tr(⌈ρ⌉)→Par(⌈ρ⌉) figuring in the proof of Proposition 5.2 cannot be said to be paradoxical in the sense expressed by Par, it can still non-trivially be said to be paradoxical in a stronger sense expressed by a new predicate $Par_{1}$. And so on (and similarly for Un). It might then be insisted that, as Field puts it in a related context, this ‘would not nearly have the devastating impact on our reasoning a stratification of truth predicates would have’ [2013: 22].

Field’s strategy has been criticized in a number of places (for example, Priest [2007], Rayo and Welch [2007], and Welch [2008, 2014]). Here we limit ourselves to noticing, first, that paradoxicality and unparadoxicality appear to be just as central as truth in the revisionary theorist’s cognitive life. That *λ* entails absurdity if reasoned with classically, and is therefore paradoxical in our sense, is a minimal but key revisionary lesson of the Liar Paradox. Second, the arguments that are usually put forward against non-hierarchical accounts of truth apply equally to paradoxicality and unparadoxicality. For instance, if ϕ’s paradoxicality-in-*S* can only be asserted by means of a stronger paradoxicality predicate ‘paradoxicality_1_-in-*S*’, it might be argued, following Kripke [1975: 695–6], that there is no way to interpret a discourse in which two speakers attribute paradoxicality-in-*S* to everything that they say.

## 8 Concluding Remarks

Non-classical approaches to paradox are attractive, for two main reasons: they allow one to retain extremely intuitive naïve semantic principles; and they often allow one to do so by using non-classical logics that can be both natural and strong.^42^ This is a tempting, if ultimately radical, thought. Existing revisionary approaches cannot express one of the basic lessons of the semantic paradoxes (namely, that certain sentences trivialize one’s theory if reasoned with classically, while others don’t)—facts that are built into the classical recapturing properties enjoyed by each of the representative theories discussed in this paper. As a result, revisionary theorists must resort to logics that are significantly weaker than the four families of logic introduced in section 4. This is especially problematic for revisionary theorists who place special emphasis in their theories’ ability to recapture classical theories and to restrict classical logic exactly when it creates paradox-driven trouble. The original Liar Paradox, and other run-of-the-mill paradoxes, can be blocked by weakening classical logic. But, in view of the paradoxes of paradoxicality and unparadoxicality, the Liar Paradox inevitably re-emerges in new theory-relative clothes to exact its revenge.^43^

## Footnotes

1 See, e.g., Priest [2006a], Field [2008], Beall [2009], Zardini [2011], and Cobreros et al. [2012].

2 See, e.g., Field [2008: ch. 13] and Beall [2009: sec. 1.1].

3 See, e.g., Priest [2006a: 221], Beall [2009: 111–12], Zardini [2011: 518–19], and Woods [forthcoming].

4 See, e.g., Gauker [2006: sec. 3], Leitgeb [2007], Rayo and Welch [2007], and Welch [2014].

5 See, e.g., Priest [2006b: ch. 5], Field [2008: sec. 21.1], and Beall [2009: ch. 3]. The Boolean negation ¬ϕ of ϕ takes value 1 whenever ϕ has a value *other than* 1.

6 See, e.g., Field [2008: 356] and Beall [2009: 39, 57].

7 See, e.g., Ripley [2012, 2013: 146].

8 A *multiset* is just like a set, except that repetitions count. We use {} as brackets for sets, and [ ] as brackets for multisets. Thus, {ϕ,ψ,ψ} and {ϕ,ψ} are the same set, but [ϕ,ψ,ψ] and [ϕ,ψ] are distinct multisets. We omit brackets from multisets in sequents—e.g. writing ϕ,ϕ⊢ψ instead of [ϕ,ϕ]⊢ψ.

9 This suffices for the purposes of this paper: the results in section 5 only require propositional logical rules. For simplicity, we have opted for a single-conclusion natural deduction calculus in sequent-style in which structural rules are explicitly formulated.

10 A double line indicates that a rule can be read in both directions.

11 More precisely, λ is the sentence $\neg Tr(t_{\lambda })$, where $t_{\lambda }$ is a closed term such that $t_{\lambda }=\lceil \neg Tr(t_{\lambda })\rceil$. However, in the theories that we consider, λ and $\neg Tr(t_{\lambda })$ are always intersubstitutable, and we will therefore stick to this simpler formulation. The same goes for the other ‘self-referential’ sentences to be introduced later.

12 Triviality can also be established, without making use of ⊥-E, via Curry’s Paradox. The paradox involves a sentence γ identical to Tr(⌈γ⌉)→ψ (where ψ is any sentence). Given SRef, SContr, Cut, →-I, and →-E, a Liar-like argument allows one to ‘prove’ ψ.

13 Examples of classical hierarchical treatments include Tarski [1936], Parsons [1974], Glanzberg [2004], and Murzi and Rossi [2018]. For classical non-hierarchical approaches, see, for example, Feferman [1991], Leitgeb [2005], and Halbach [2011: secs. 19.3–19.5].

14 The revisionary literature to date has focused almost exclusively on theories validating SRef. While it is possible to devise a revenge paradox for SRef-free approaches along the lines of the ones to be developed in section 5, we don’t give the argument here, for reasons of space. For a recent proposal involving a restriction of SRef, see Nicolai and Rossi [2018].

15 Paracomplete theories have been developed in Kripke [1975], Halbach and Horsten [2006], Field [2008, 2013], and Horsten [2011]. For paraconsistent logics and their application to semantic paradoxes, see, e.g., Asenjo [1966], Priest [1979, 2006a], Goodship [1996], and Beall [2009].

16 See, e.g., Shapiro [2011a], Mares and Paoli [2014], and Zardini [2011].

17 Zardini’s full theory $IKT^{\omega }$, as he calls it, includes some controversial infinitary rules for the quantifiers [Zardini 2011: 508]. The revenge paradox to be developed in section 5.5 applies not only to Zardini’s full theory, but also to a version of his theory in which the conjunction and disjunction operators are additive—namely, governed by context-sharing rules—and the quantifiers are governed by standard, finitary rules (for discussion, see Zardini [ibid.: 509–10]).

18 See, e.g., Weir [2005], Ripley [2012], and Cobreros et al. [2013].

19 See, e.g., Priest [2006a: 221], Field [2008], Beall [2009: 111–12], and Zardini [2011].

20 Field’s quote is, strictly speaking, misleading: current non-classical approaches to semantic paradox seek to preserve classical logic also for unproblematic uses of the truth predicate, such as ‘All the theorems of Peano Arithmetic are true’, ‘If 65+57=125 is true, then 65+57≠125 is not true’, and so on.

21 Our characterisation of classical recapture in LP is very much in line with an account discussed in Priest [2006a: 117–18] and Beall [2011].

22 We are adapting Theorem 3.19 of Zardini [2011] to our framework.

23 We should mention at least one alternative proposal for recapturing classical logic within a non-classical theory—Priest’s *minimally inconsistent* LP, or mLP for short [2006a: 222ff). mLP is a non- monotonic logic that behaves like classical logic in the case of arguments with consistent premises and behaves like LP in the case of arguments with inconsistent premises. As far as we know, mLP has not been axiomatized. Whether our account of classical recapture can be extended to mLP depends on whether such a logic can be given an axiomatisation satisfying **Classicality Principles**.

24 For recent discussion of revenge, both object-linguistic and meta-theoretic, see, e.g., Beall [2007a], Field [2007], and Shapiro [2011b]. For general background on revenge, see the essays in Beall [2007c] and Scharp [2013: ch. 8].

25 See, e.g., Field [2008: sec. 21.1] and Beall [2009: sec. 3.4].

26 Field’s [2007] theory involves a hierarchy of ever-stronger notions of indeterminacy, whereby sentences such as λ can be said only to be indeterminateα, for some level α in the hierarchy.

27 Our notion of paradoxicality-in-*S* is closely related to Beall’s [2015] notion of a *trivializer* for *S*.

28 Since the chosen theory *S* will always be clear from context, we simply write Par(x) for paradoxicality and Un(x) for unparadoxicality.

29 For a general case for naïve truth principles, see Field [2008: 209ff.] and Beall [2009: sec. 1.1].

30 With the exception of non-contractive theories, all of the rules for Par and Un presented in this paper can be given a context-sharing formulation without affecting our revenge paradoxes.

31 To our knowledge, higher-order rules were first introduced by Schroeder-Heister [1984: 1284–5], who pointed out that if sentence-assumptions are technically *temporary axioms* then nothing should prevent one from also making use of *rule-assumptions*, understood as *temporary rules* (in our setting, such temporary rules are sequent-assumptions).

32 See Tennant [2012: 4–5].

33 The derivation is also non-normal in the sense of Tennant [2012], since the major premise of Cut−Un−E in the last step of the derivation has non-trivial proof work above it.

34 Core Logic is a non-transitive logic, all of whose proofs are in normal form. The logic comes in a constructive and in a non-constructive variety; our remarks apply to both. As Tennant [2015] observes, the derivations of semantic paradoxes such as the Liar are all invalid in Core Logic supplemented by suitable rules for naïve truth. More precisely, just as in ${STTT}_{0}^{+}$, the final use of Cut in the paradoxical derivations (or of the unrestricted ¬-E and →-E) is disallowed, on the ground that it would bring in non-normality. Although Tennant doesn’t prove consistency for a theory of naïve truth whose underlying logic is Core Logic, it can be shown that the consistency proofs available for STTT [Ripley 2012; Cobreros et al. 2013] immediately extend to the theory given by closing Core Logic under the rules for naïve truth (this follows from the fact that Core Logic is a relevant subsystem of the logic ST). This in turn answers the technical question raised by Tennant [2015: sec. 4.3.4] of whether one can prove the non-triviality of the approach that he recommends.

35 For reasons of space, we leave out the precise formulation of the template.

36 Standard provability predicates satisfy the Hilbert-Bernays ‘derivability conditions’, i.e. predicate analogues of the rules of necessitation and of the K and 4 axioms of modal logic.

37 For more discussion, see Field [2017] and Murzi and Rossi [forthcoming].

38 Such a restriction is also inadequate to fulfil the expressive role of ‘true’. Suppose that one tried to express her acceptance of all of PA’s theorems by asserting that all of the theorems of PA are true. If ‘true’ is modelled by a $\Sigma_{n}$-truth predicate, one would have thereby only expressed acceptance of the $\Sigma_{n}$-theorems of PA.

39 For instance, classical theories do not derive the following instance of the T-Schema: Tr(λ)↔λ.

40 See, e.g., Priest [2006b: ch. 5], Field [2008: sec. 21.1], and Beall [2007b, 2009: ch. 3].

41 The strategy can be generalized to other non-classical approaches [Field 2008: ch. 27].

42 Leon Horsten [2009, 2011] argues for the naturalness of a certain non-classical theory of truth. Field [2008], Zardini [2011], Ripley [2012], and Cobreros et al. [2013] emphasize the logical strength of their respective approaches.

43 Early versions of some of this material were presented at conferences and colloquia in Amsterdam, Barcelona, Bochum, Groningen, Oxford, Rome, Florence, Montreal, and Pamplona. We thank these audiences for valuable comments. We also wish to thank Jc Beall, Andrea Cantini, Paul Egré, Hartry Field, Rosanna Keefe, Francesco Paoli, Lavinia Picollo, Dave Ripley, Lucas Rosenblatt, Lionel Shapiro, Neil Tennant, and Elia Zardini for helpful discussions over the years. Special thanks to Dave Ripley for invaluable comments on previous drafts of this paper that have led to substantial improvements, and to Leonie Eichhorn and Sascha Selke for much-needed editorial assistance.

## Appendix

We provide proofs of Propositions 5.2, 5.4, 5.6, and 5.8.
**Proposition 5.2.** K3TTP *is trivial, and so is the closure under* LEM-Par-I *and* LEM-Par-E *of any theory extending* $K3TT^{+}$ *.*
*Proof.* We make use of the following $K3TT^{+}$-valid form of →-I (given →’s materiality, this is in effect a restricted form of ∨-I):

We now reason thus, in K3TTP. Let ρ be identical to Tr(⌈ρ⌉)→Par(⌈ρ⌉). We first prove that ρ∨¬ρ⊢Tr(⌈ρ⌉)∨¬Tr(⌈ρ⌉):

Call this derivation $D_{0}$. In our next step, we prove that ρ∨¬ρ⊢ρ:

Call this derivation $D_{1}$. We use it to show that ρ is paradoxical:

We now have a proof of Par(⌈ρ⌉)—call it $D_{2}$. This in turn yields absurdity, as the following derivation shows:

**Proposition 5.4.** LPTTU *is trivial, and so is the closure under* LNC-Un-I *and* LNC-un-E *of any theory extending* $LPTT^{+}$ *.*
*Proof.* Let ς be a sentence identical to ¬Tr(⌈ς⌉)∧Un(⌈ς⌉). We reason in LPTTU. We begin by proving ς∧¬ς⊢⊥:

Call this derivation $D_{0}$. We can use it to prove ¬ς:

Call the above derivation $D_{1}$. Together with $D_{0}$, it yields a proof of triviality.

**Proposition 5.6.** MALLTTP *is trivial, and so is the closure under* LC*-*Par*-*I *and* LC*-*Par*-*E *of any theory extending* $MALLTTP^{+}$ *.*
*Proof.* Let ρ be the sentence Tr(⌈ρ⌉)→Par(⌈ρ⌉). We reason in MALLTTP. We first prove ρ, on the assumption that ρ satisfies ρ→(ρ∧ρ):

Call this derivation $D_{0}$. We use it to prove that ρ is paradoxical from two occurrences of ρ→(ρ∧ρ):

Call the above derivation $D_{1}$. We now use it to prove that ρ is paradoxical:

Call the above derivation $D_{2}$. It yields a proof of ⊥, and hence of the triviality of MALLTTP:

**Proposition 5.8.** $STTTU_{0}$ *is trivial, and so is the closure under* Cut-Un-I *and* Cut-Un-I *of any theory extending* ${STTT}_{0}^{+}$ *.*
*Proof.* We reason much in the same way as in the paraconsistent case. We reason in $STTTU_{0}$. We let ς be identical to ¬Tr(⌈ς⌉)∧Un(⌈ς⌉) and prove ς⊢⊥:

Call this derivation $D_{0}$. We can now assume ⊢ς, use the conclusion of $D_{0}$ (namely, ς⊢⊥) to derive ⊢⊥, and finally discharge our assumptions and categorically conclude that ⊢Un(⌈ς⌉) via Cut-Un-I:

Call this derivation $D_{1}$. $D_{0}$ and $D_{1}$ can finally be combined to yield a proof of ⊥:
